# Virtual reality exposure therapy to decrease anxiety before surgical invasive procedures in hemodialysis patients: an interventional study

**DOI:** 10.1186/s12882-024-03461-w

**Published:** 2024-01-24

**Authors:** Tahereh Hosseini, Manijeh Hooshmandja, Morteza Noaparast, Rita Mojtahedzadeh, Aeen Mohammadi

**Affiliations:** 1 Department of E-Learning in Medical Education, Center of Excellence for E-learning in Medical Education, School of Medicine, Tehran University of Medical Sciences, Dolatshahi Alley, Naderi St, Keshavarz Blvd, 14166-14741 Tehran, Iran; 2 Department of Educational Technology in Medical Sciences, Smart University of Medical Sciences, Tehran, Iran; 3 Department of General Surgery, School of Medicine, Prehospital and Hospital Emergency Research Center, Imam Khomeini Hospital Complex, Tehran University of Medical Sciences, Tehran, Iran; 4 Health Professions Education Research Center, Tehran University of Medical Sciences, Tehran, Iran

**Keywords:** Renal dialysis, Anxiety, Virtual reality, Surgical procedures, Operative, Therapeutics

## Abstract

**Background:**

Hemodialysis patients are prone to anxiety and depression due to physiological changes and psychological tensions that leave irreversible effects on the patients. In this regard, the present study was an attempt to provide a simulated situation by using virtual reality on the anxiety level of dialysis patients before performing surgical procedures.

**Methods:**

This non-equivalent groups pre-posttest quasi-experimental study was conducted in 2022 in a hemodialysis ward of Imam Khomeini General Hospital affiliated with Tehran University of Medical Sciences. The study population was patients suffering from kidney failure referred to this hospital. The participants were 30 patients selected from the study population, who were allocated into two groups (15 in each experimental and control group). The control group received routine training in the ward. The experimental group participants watched five educational virtual reality (VR) contents in addition to the routine training. They used head-mounted display VR (VR BOX headset 2.0 virtual reality glasses) to immerse in a virtual environment similar to the real world covering the experience of entering the operating room, during the surgery, and after surgery. The data gathering instrument was a valid and reliable anxiety inventory. The collected data was analyzed at a significance level of 0.05.

**Results:**

The study findings indicated that the anxiety scores of the experimental and control groups had no significant difference before intervention. However, after the intervention of virtual reality, the state and trait anxiety of the experimental group participants were significantly lower than the control group (*P*-value < 0.01).

**Conclusion:**

The application of VR for maintenance hemodialysis patients before invasive surgical procedures decreases patients’ anxiety. Considering the devastating and undeniable impacts of anxiety on the lives of patients referring to hemodialysis centers, the application of VR is suggested to decrease their anxiety.

## Background

Anxiety is a pervasive, unpleasant, unknown, vague feeling of fear and worry with an unknown origin that is accompanied by physiological changes such as palpitation, perspiration, headache, and shortness of breath [1]. Anxiety-related disorders originate from mental factors [2] that can be brought on by exposure to new situations, such as those in medical settings [3]. Various factors, including age and prior experience, affect how people react to anxiety [4].

Hemodialysis is a stressful process that induces numerous social, economic, and cultural changes in the patient’s life, which lead to psychological (or mental) disorders including depression and anxiety. Studies indicate that the prevalence of depression and anxiety in these patients is 20–70% and 30–60%, respectively [5]. Hemodialysis patients are aware of the deteriorative nature of their disease and suffer from the stresses of exhausting therapeutical measures including dialysis. On the other hand, the chronic nature of the disease adversely affects the mental and social functions of the patients and leads to increased morbidity, frequent hospitalization, increased cost of disease, and mortality [6]. Likewise, literature shows that in other medical conditions like stroke, anxiety prevents adherence to dietary regimens, and prescribed treatments, and adversely affects self-care and therapy results; however, with mild interventions, it could be relieved [7, 8].

There are two types of anxiety including state and trait ones. State anxiety is defined as an unpleasant emotional response while coping with threatening or dangerous situations [9], which includes a cognitive appraisal of a threat as a precursor for its appearance [10]. On the other hand, trait anxiety refers to stable individual differences in a tendency to respond with an increase in state anxiety while anticipating a threatening situation. Spielberger characterized trait anxiety as a general disposition to experience transient states of anxiety, suggesting that these two constructs are interrelated [11].

“Virtual Reality (VR) exposure treatment” is among the exciting and advanced emerging technologies that is potentially useful to treat anxiety and depression [12]. VR uses real-time multisensory channel stimulations, mainly visual and auditory. The person is placed in a virtual environment similar to the real world that is free from real-world risks. This interactive new technology creates a sense of being in a physical environment and facilitates interaction with the environment by emphasizing the graphical aspects [13]. In this technology, three factors of interaction, imagination, and immersion are influential in simulating the environment. Among these factors, “interaction” refers to the mutual relationship between the person and the virtual world that is focused on the optimization of user control [14]. Based on interaction, responsiveness, and reaction to the information, the VR platform creates mutual communication between users and computers [15]. According to this mutual interaction, cognitive learning improves. Meanwhile, “imagination” implies how the user feels a real presence in a virtual world despite the awareness of the presence of the physical body in another world. “Immersion” indicates the amount of the user experience of presence in virtual worlds; in other words, disconnection with the physical world or immersion in the virtual world [16].

Patient training via VR attracts their attention and focus toward the simulated virtual world of therapeutic team members and provides an environment for a sense of presence formation through involvement with images, colors, voices, touch-visual and auditory pads, and creating motions and real physical activities [17]. The sensation of being in a real situation is the major prerequisite to understanding diverse emotions in therapeutic processes. For example, renal failure patients who experience anxiety in virtual spaces can show the appropriate reactions to control it [18].

Some psychologists believe that a large number of patients who cannot achieve meaningful recovery via classic approaches over time might be treated by real therapeutic exposures. In other words, by VR time machine, and experience of the future spaces while being exposed to new events (such as severe pain during dialysis venipuncture), they suffer less and it supports them to manage and control their mental pressures and tensions related to unexpected events [19]. Hence, this research aimed to evaluate the effect of using VR contents on anxiety in hemodialysis patients undergoing surgical interventions.

## Methods

This is a nonequivalent-groups pre-posttest quasi-experimental study that was conducted in April-June 2022.

### Research setting and participants

The research setting was Imam Khomeini General Teaching Hospital, located in Tehran. The hospital has 1500 active beds with an independent hemodialysis ward. The study population was all patients afflicted with kidney failure who were referred to the hospital during the research period (*N* = 170) and underwent dialysis catheter surgery. The study participants were 30 hemodialysis patients (15 in each experimental and control group). We allocated the first 15 admitted patients to the control group and then underwent the process for the intervention group to avoid the contamination bias. It was infeasible to deliver the VR contents to some of the patients admitted to the ward at the same time and asking the others not to watch it.

The inclusion criteria were written informed consent, lack of underlying diseases such as neurological and mental disorders based on the patients’ medical records, being literate, and no use of anti-depressive drugs or tranquilizers. Exclusion Criteria included dissatisfaction with continuing to participate in the study and absence in the training sessions.

In this study, the Persian version of Spielberger’s State-Trait Anxiety Inventory (STAI) [9] which was standardized in 1993 in Iran [20] was used. In this scale, the purpose is to measure the state anxiety level from low to high. It means that low scores indicate peace of mind, moderate scores indicate medium tension and worry, and high scores reflect intense anxiety close to fear and panic. In this inventory, participants express the intensity of their feelings on a 4-point Likert scale of “not at all”, “somehow”, “moderate”, and “very much” at a certain time. Also, they express how they felt in general as their trait anxiety on a 4-point Likert scale of “rarely”, “sometimes”, “often and “almost always”. The first 20 questions of State Anxiety could be considered as a section of the life of a person that happens in response to specific tension-provoking situations and measures the feelings of the person at the time of responding to the questions. The following 20 questions evaluate individual differences in response to stressful situations with different levels of stress or indicate general or normal feelings. In this study, the internal consistency of the inventory according to Cronbach’s Alpha was 0.964.

### The creation of VR educational contents

To prepare the VR content videos were recorded about the real experience of all the events and procedures that patients undergo while admitted to the ward, entering the operating room, during the surgery, and after surgery. Then, these experiences were narrated as scenarios that covered the step-by-step events without any exaggeration or understatement in a completely real and natural way by the experts. All the scenarios were reviewed and confirmed by three nephrology experts who were not among the research team members. VR contents were developed based on these scenarios. The content of the videos was combined with the voices heard in the operating room, such as the device alarm, and the words and phrases expressed by operating room staff and surgeons. All the videos were developed with Samsung Gear 360 camera, making it possible to create a true virtual reality experience. The patients and the staff who participated in the videos were all healthy staff of the hospital who had the experience of working in hemodialysis wards. After training, they played voluntarily in videos as simulated patients and staff. The whole content was divided into five separate sessions (each one about six minutes). The contents of VRs were as follows:

- VR content 1: A dialogue happened between the patient and the accompanying nurse in the ward before going to the operating room to brief the patient about the procedure and answer the frequently asked questions. This part ended with showing these two going toward the operating room.
- VR content 2: The content simulated the operating room and a dialogue happened among the accompanying nurse, the operating room nurse, and the patient showing the devices and explaining the surgery procedure; ending with the patient being transferred to the operating room bed.
- VR content 3: In this content the surgeon was added to the team and the previous dialogue continued with more detailed questions of the patient. Then, the surgery was started with the patient’s anesthesia procedure. The content depicted the surgery and ended with the patient being transferred to the recovery room.
- VR content 4: This part started with the patient in the recovery room and depicted what would happen while becoming conscious. It ended with the patient being transferred to the ward.
- VR content 5: In this part, the post-operation events were simulated. The patient was briefed about the conducted procedure through a dialogue with the nurse. In addition, the post-operation care in the ward was depicted. Finally, the nurse trained the patient about self-care and follow-ups. It ended with the patient being discharged from the hospital.

These contents and the VR software were uploaded and installed on a mobile phone which was placed over the VR glasses (VR BOX headset 2.0 virtual reality glasses, Bnext, China). So, the patient could watch the videos played on the phone. Therefore, with VR glasses that were put over the patients’ eyes and headphones in their ears, they entered the operating room virtually in such a way that the patient was stable and was tracked by the head movements. Each participant watched all five VR contents during the day before the surgery at her or his convenient pace.

### Research implementation

To conduct the study, approval was obtained from the ethics committee of the Tehran University of Medical Sciences (TUMS) (IR.TUMS.MEDICINE.REC.1400.1035). To follow the rules of ethics, permission to conduct the research was taken from the hospital authorities and informed consent was obtained from all the study participants.

There was no compulsion for the study participants to participate in the study till it ended. Besides, the study participants were informed that nonparticipation in the research causes no harm to them, and conducting the research does not interfere with the patient’s treatment process.

At first, the importance and necessity of conducting the study were explained to the study participants. After obtaining the informed consent, the participants of both experimental and control groups were asked to complete the anxiety inventory (before intervention). Completing the inventory was self-administered, and in cases where the participant was unable to complete the inventory, the researcher completed it by asking questions orally. Then, participants in both groups received the routine information from the ward nurse orally one day before going to the operating room. Besides, participants in the experimental group received the necessary information about the surgery via VR technology (five VR contents) individually on the same day too. Meanwhile, it took a total of two months to recruit the sample size for both study groups. The interval between receiving the inventory before and after the intervention was about eight hours for both groups.

SPSS (IBM Corp. (2020). IBM SPSS Statistics for Windows, Version 22) [Computer software]. IBM Corp.) was used for data analysis. The IRCT registration number is IRCT20220416054545N1, registered on 26/04/2022, and the registered trial name is “Evaluation of the effect of training using virtual reality on reducing anxiety before performing invasive surgical procedures in patients with maintenance hemodialysis.”

## Results

The study participants were 15 in each of the experimental (7 females and 8 males) and control groups (5 females and 10 males). The participants were 25 to 62 years old and the average ages of the participants in the experimental and control groups were 40.7 (12.82) and 40.8 (14.19) respectively. There were no significant differences according to their sex (Chi^2^ = 02.400, *P*-value = 0.12) and age (T = 0.27, *P*-value = 0.98) between the study groups. Table 1 includes the characteristics of the study participants.

**Table 1:** Characteristics of the study participants

Categories	Intervention	Control	Sig
Sex: N			
Male/Female	8 / 7	10 / 5	0.12^*^
Age: Mean (SD)	40.7 (12.82)	40.8 (14.19)	0.98^**^
Degree			
Not completed high school diploma: N (%)	8 (53.3)	4 (26.7)	0.19^*^
High school diploma: N (%)	5 (33.3)	5 (33.3)	
University degree: N (%)	2 (13.3)	6 (40)	
History of previous surgery (yes/no)	7 / 8	6 / 9	0.71^*^

* Pearson Chi-square test, ** T-test

All the patients allocated to the study groups completed the inventory. There was a significant difference between State and Trait Anxiety before and after intervention in the experimental group (*p*-value < 0.01). There was no significant difference between State and Trait Anxiety before and after intervention in the control group with *p* = 0.67 and *p* = 0.49 respectively (Table 2).

**Table 2:** Comparison of the State and Trait Anxiety scores between groups

Study Group		Pre-test	Post-test	*P*-Value^*^	ANCOVA (*P*-Value)
		Mean (SD)			
State Anxiety scores	Control	58.33 (12.19)	58.34 (9.25)	0.67	< 0.01
	Intervention	62.53 (12.76)	36.73 (9.21)	< 0.01	
	Sig. ^**^	0.365			
Trait Anxiety scores	Control	59.60 (9.13)	58.73 (9.46)	0.49	< 0.01
	Intervention	64.53 (10.0)	35.53 (11.27)	< 0.01	
	Sig. ^**^	0.169			

^*^ Paired T test, ^**^Independent T test

In the next step, we compared the levels of State and Trait Anxiety after intervention between the experimental and control groups. To equalize the pre-test scores in both groups, ANCOVA was used. The groups were entered into the equation as the independent variable, the post-test scores as the dependent variable, and the pre-test scores as the covariance. Regarding the post-test results, the State and Trait anxiety scores in the experimental group; i.e. (36.73 (9.21) and 35.53 (11.27) respectively, had a significant difference with the same anxiety scores in the control group; i.e. 58.34 (9.25) and 58.73 (9.46) respectively (Table 2). As a result, it could be concluded that the patients who received VR training suffer from less State and Trait Anxiety compared to those who did not.

## Discussion

Recently, VR has received much attention as a new approach to treating anxiety disorders [21]. Due to the intense undesirable psychological and physiological effects of anxiety on patients, it must not be ignored. The psychological effects of anxiety include feelings of discomfort, panic, worry, tension, apprehension, catastrophizing, or obsessive thinking [22]. Hence, this study was an attempt to investigate VR effectiveness in reducing anxiety before invasive surgical procedures in hemodialysis patients. The experimental and control groups data were collected by completing the valid and reliable anxiety inventory.

The results of this study indicated that the anxiety levels of patients trained with VR contents in the experimental group were significantly lower than the control group after the educational intervention. This finding is consistent with the findings of Ioannou et al. [23], Piskorz & Czub [24], Zeng et al. [25], Baus et al. [26], Baños et al. [27], and Schneider et al. [21]. In research conducted by Zeng et al. [26] on the effectiveness of VR, they found that VR helps to reduce signs and symptoms of anxiety and depression. In a study conducted by Shah et al. [28] in Singapore to determine the effects of a VR program on tension management in people with mood disorders it was found that after completion of the program, the level of anxiety and depression significantly decreased. Baños et al. [27] in their research indicated that VR exposure therapy was used to create positive feelings in hospitalized patients. Another study on the effectiveness of VR exposure therapy in reducing tension and intensity of venipuncture pain in pediatric clinic patients showed improvement and reduction of pain and tension [24]. Moreover, Schneider et al. [21] found that there was a sharp reduction in anxiety symptoms after chemotherapy in patients who received VR exposure therapy during chemotherapy, which is consistent with the findings of this study.

In addition, Shah et al. [28] study indicated the effectiveness of virtual reality exposure therapy in reducing stress and depression in patients suffering from mood disorders. Donnelley et al. [29] indicated that head-mounted VR is a valuable device for occupational therapy to simulate the environments in which patients with anxiety disorders participate. Evoking presence via multi-sensory features and body representation may enhance the effectiveness. In virtual reality, a computer program creates an anxiety-provoking virtual environment, by merging graphics, visual displays, graphic games, body tracking instruments, and other sensory input devices [30].

According to the theory presented by Borcovic, anxiety is related to lower levels of mental imagery and acts as a factor to avoid unpleasant mental imagery. Therefore, according to the principles of this theory, exposure could be a successful treatment for these disorders that can be used in natural environments or the form of imagery exposure [31]. VR via displays or special images, creates a real visual experience for the users because the visual aspect is one of the most important features of virtual environments. Therefore, the user feels like being in a real environment. These environments enable people to repeat and expand experiments in the real world in a controlled environment [19]. The level of the user’s experience of presence as “being in the place” or “immersion” in the virtual environment confirms the role of virtual reality in reducing the level of psychological disorders.

The attractive VR environment and background [32], lack of fear of failure and despair [33], motor imagery and action observation [34], and reduction in the number and time of VR sessions compared to traditional treatment methods [32] are among the positive features of virtual reality that can increase motivation, encourage a person, and increase self-confidence, and control of treatment conditions [33].

In addition, patients’ distraction and preoccupation while working with VR, distracts them from disease and its related problems, and can reduce their anxiety [35]. In a VR environment, patients confront the most anxiety-provoking situations and by repeating the situations, they become desensitized. In other words, the patient faces her or his fears and there is no need to mentally visualize the scene of fear.

This study had some limitations. The study design was not a randomized experimental one and had a modest sample size. Therefore, the findings need to be interpreted carefully. In addition, there were imbalances between the intervention and control groups regarding sex and education. Although these differences were not statistically significant, this may be because of the small sample size and is recommended to be considered in future studies.

## Conclusion

According to the study findings on the effectiveness of VR on anxiety reduction before invasive surgical procedures in hemodialysis patients, VR as an interventional therapeutic measure is recommended to reduce patients’ psychological problems including anxiety. This study had some limitations. There were environmental and psychological factors that could affect patients’ anxiety and were not under the control of the researcher. Considering the nature of educational content observation via 3D glasses and its adverse effects (e.g., dizziness and nausea), viewing the educational content continuously with a high number of sessions was impossible.

It is recommended to conduct future studies to compare VR exposure therapy and other common treatments such as cognitive-behavioral therapy and mindfulness-based stress reduction. Finally, it can be concluded that despite little scientific experience with this method, we can achieve promising and efficient results for stress reduction and to achieve the highest efficiency it must be investigated extensively in future research.

## Data Availability

The datasets used and/or analyzed during the current study are available from the corresponding author upon reasonable request.

## Abbreviations

VR: Virtual Reality
STAI: State-Trait Anxiety Inventory
3D: Three dimensional
TUMS: Tehran University of Medical Sciences
ANCOVA: Analysis of Co-Variance
IRCT: Iranian Registry of Clinical Trials
